# To Restore Drowning Persons

**Published:** 1893-02

**Authors:** 


### HOW TO RESTORE DROWNING PERSONS.

Every body may be called upon at any season of the year to afford assistance to drowning persons while the doctor is being sent for, and Professor Laborde's simple method for restoring breath when all other means have failed deserves to be universally known. A Paris correspondent tells us that the other day, at a watering place in Normandy, two bathers, a young man and a boy, who were unable to swim, went out of their depth and disappeared. They were brought on shore inanimate, and were taken to the village. Two doctors were sent for, but the young man gave no sign of life, and they declared he was dead. M. Laborde, who was fishing at half-an-hour's distance, came up as soon as he heard of the accident. He examined the body and found that the extremities were cold and the heart had stopped. Then, taking hold of the root of the tongue, he drew it violently forward, giving it a succession of jerks in order to excite the reflex action of the breathing apparatus, which is always extremely sensitive. At the end of a few minutes a slight hiccough showed that the patient was saved. In addition to the usual restorative means, Professor Laborde, in extreme cases, rubs the chest with towels soaked in hot and nearly boiling water, although the skin is blistered by this.

